# Case report: Dual dabrafenib and trametinib therapy for treating BRAF V600E mutated lung adenocarcinoma with BRCA2 germline mutation post multiline progression

**DOI:** 10.3389/fonc.2024.1387388

**Published:** 2024-04-23

**Authors:** Huimin Zhang, Xiaofeng Cong, Jiaxin Yin, Chen Chen, Ziling Liu

**Affiliations:** Cancer Center, The First Hospital of Jilin University, Changchun, China

**Keywords:** dabrafenib, trametinib, BRAF V600E mutation, BRCA2 germline mutation, lung adenocarcinoma

## Abstract

The v-raf murine sarcoma viral oncogenic homolog B1 (BRAF) V600E is a rare mutation that functions as an oncogenic driver in patients with non-small cell lung cancer (NSCLC) leading to the overactivation of the RAS-RAF-MEK-ERK (MAPK) pathway and the subsequent uncontrolled cell proliferation. Understanding the mechanism behind BRAF mutation, its inhibition, and relationship to the upstream and downstream effector is essential for advancing treatment strategies for NSCLC patients with the BRAF V600E mutation. Next-generation sequencing studies have identified the presence of breast cancer susceptibility gene 1/2 (BRCA1/2) mutations in NSCLC patients, which are pathogenic variants associated with breast, ovarian, and prostate cancers. Although poly ADP-ribose polymerase (PARP) inhibitors are currently an approved treatment option for malignant tumors linked to BRCA1/2 pathogenic variants, the therapeutic potential of PARP inhibitors in NSCLC remains unclear. The development of genetic testing provides a platform for investigating the pathophysiological mechanisms of genetic mutations above. Here, we report a novel case of a middle-aged non-smoking female diagnosed with BRAF V600E and BRCA2 germline mutated lung adenocarcinoma, who had previously undergone a diverse array of cancer-targeted therapies, including PARP inhibitor, before the identification of the BRAF V600E mutation. Following this, a combination of dabrafenib and trametinib was administered and induced a rapid and positive response within two months. Our case not only highlights the importance of dynamic and repetitive genetic testing in managing patients, but contributes to the growing body of clinical evidence supporting the efficacy of BRAF/MEK co-inhibition in patients harboring a BRAF V600E mutation and provokes thinking for further research into the impact of PARP inhibitors in BRCA1/2-mutated NSCLC.

## 1 Introduction

The pathway of RAS-RAF-MEK-ERK transmits signals through sequential activation, enabling the transduction of signals from the cell surface into the intracellular space, where it plays a pivotal role in regulating multiple crucial physiological processes such as cellular proliferation and survival programs (1). RAS protein family, including KRAS, HRAS and NRAS, functions as a molecular switch by cycling between active guanosine triphosphate (GTP) - bound states and inactive guanosine diphosphate (GDP) - bound states (2). Aberrant activation of RAS protein leads to the development of cancer. Among RAS protein family, KRAS mutations are the most common type in NSCLC cases, which are generally mutually exclusive with other major driver mutations such as EGFR, BRAF and ALK (3). The first downstream effectors activated by RAS belong to the RAF family which comprises ARAF, BRAF and CRAF. Among them, BRAF is a proto-oncogene, encoding a serine/threonine protein kinase which is the most active isoform of the RAF family. Mutation of BRAF is present in approximately 7-8% of all solid tumors (4), and it is present in approximately 4% of all NSCLC cases (5), with about 50% classified as class I mutations, specifically the BRAF V600E mutation (6). BRAF V600E mutation tends to manifest in mild or never-smoking women and has been associated with non-mucinous adenocarcinoma with a microgrowth pattern and strong TTF-1 expression, whereas non V600E mutation tends to exhibit a mucus component and a higher prevalence in male smokers (5, 6). Extensive research into the mechanisms of BRAF mutation has led to significant advancements in the field of targeted therapy for BRAF V600E (7). Consequently, the detection of BRAF mutations in the diagnosis process has become a routine test to identify patients eligible for precision cancer treatment. MEK1/2 are phosphorylated and activated by its upstream RAF kinases. Previous studies have indicated that KRAS or BRAF mutations are sensitive to MEK inhibitors (8). Besides, the resistance of BRAF V600 mutants to BRAF inhibitors may be caused by activating mutations of NRAS or KRAS or upregulation of receptor tyrosine kinases, which conferred sensitivity to MEK inhibition (9, 10). Therefore, the dual pharmacological inhibition of BRAF and downstream MEK has demonstrated a significant improvement in the response rate (11), leading to its establishment as the preferred first-line or back-line treatment for BRAF V600E-positive NSCLC patients (7).

BRCA1/2 are tumor suppressor genes with a pivotal role in homologous recombination (HR) - mediated DNA double - strand - break repair, and PARP inhibitors are specific drugs that target defects in the HR pathway. PARP inhibitors have shown remarkable effectiveness in treating ovarian, breast, and prostate cancer with HR defects, especially those associated with BRCA1/2 mutations. In the context of NSCLC, next-generation sequencing studies have identified the presence of BRCA1 and BRCA2 mutations in around 3% and 4.5% of the cases, respectively (12), providing a promising opportunity for targeted therapy with PARP inhibitors. However, research about PARP inhibitors is still limited, and the efficacy of PARP inhibitors in the treatment of NSCLC is still unclear.

In this report, we present a case of a lung adenocarcinoma patient with BRAF V600E and BRCA2 germline mutations. Prior to gene mutations discovery, the patient had already undergone an array of treatments, including cytotoxic chemotherapy, radiotherapy, immunotherapy, anti-angiogenic therapy, and Tyrosine Kinase Inhibitors (TKIs) therapy. The presence of germline BRCA2 mutation gave clues to treatment with olaparib while the effect was not unsatisfactory. The BRAF V600E mutation was detected during the patient’s third genetic assessment and the following administration of dabrafenib and trametinib dual therapy led to a rapid and striking reduction in tumor size. This case serves as a valuable therapeutic reference for patients with lung adenocarcinoma who have previously received extensive treatment and emphasizes the significance of ongoing and dynamic monitoring of genetic status.

## 2 Case report

**Figure 1:**
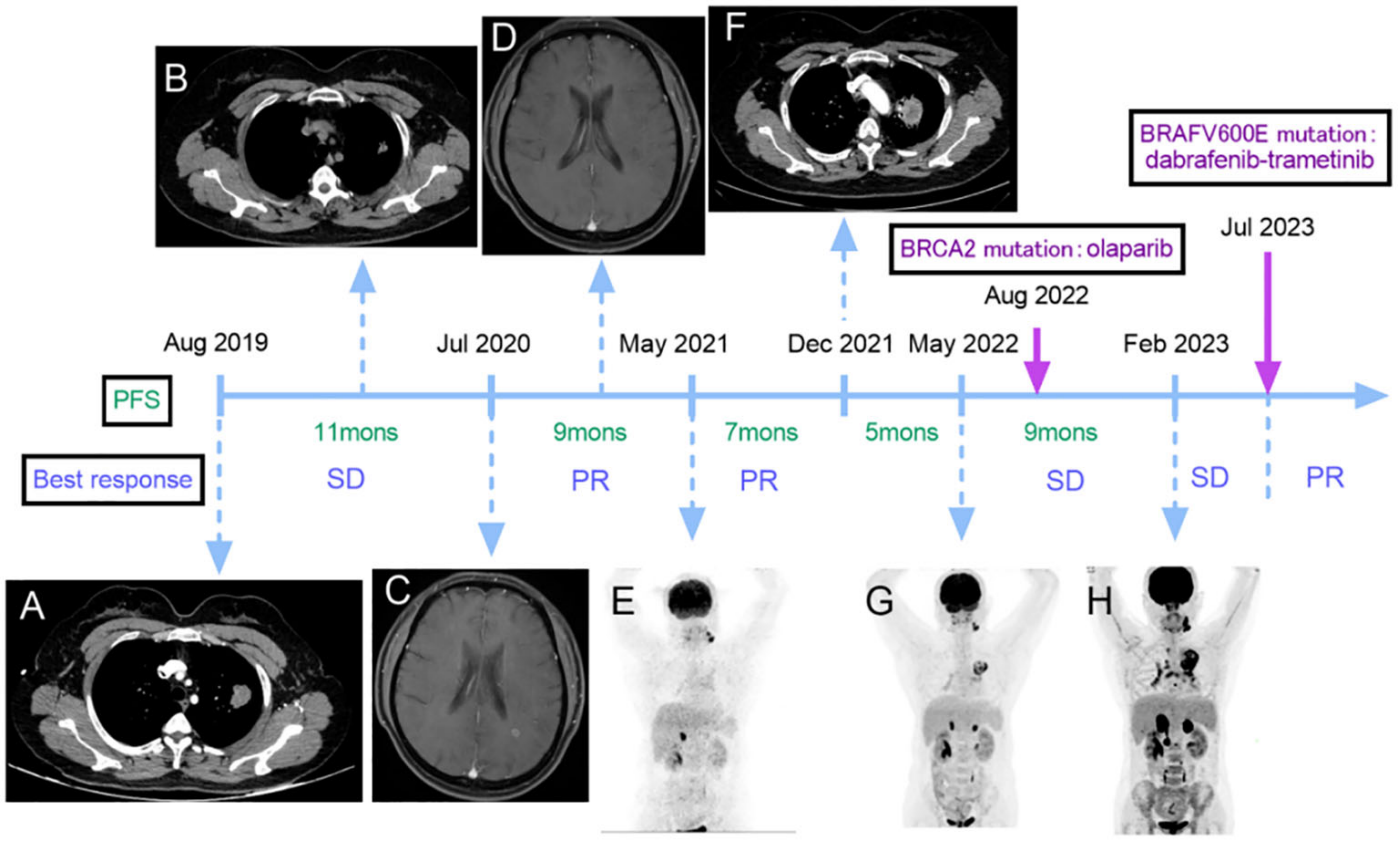
Non-small cell lung cancer (NSCLC) treatment flow chart including imaging manifestations, **(A)** lung CT revealed a dense mass that measured approximately 2.1×3.0 cm in size at initial diagnosis. **(B)** the CT scan reported that the lesion size was about 1.7×1.3 cm after the first-line treatment. **(C)** the brain MRI showed an abnormal enhancement before the second-line treatment. **(D)** no brain lesions were observed after two courses of second-line treatment. **(E)** PET-CT scan revealed metastasis in the left cervical, left submaxillary lymph nodes, and adrenal glands. **(F)** a CT scan showed an enlargement of the lesion in the superior lobe of the lung that measured approximately 4.1×3.6 cm. **(G)** a PET-CT scan disclosed that the lung lesion was about 6.5×4.4 cm, and there were 1.6 cm and 1.8 cm masses in the left and right adrenal glands, respectively. **(H)** the PET-CT scan showed new metastases in the right renal para-vascular lymph nodes and para-aortic lymph nodes. There was an increase in the size of the bilateral adrenal lesions (left: 3.9 cm, right: 4.7 cm), and the lung lesion measured 6.6×5.2 cm. The best response during per line and results of gene detections. SD, stable disease; PR, partial response.

A 48-year-old non-smoking female with no significant past medical history presented to her primary care doctor in July 2019 for three months of mild cough without blood in the sputum, and weight loss of more than five percent of body weight for the prior six months. She denied fever, chest tightness, or chest pain. The computed tomography (CT) of the chest revealed a dense mass in the superior lobe of the lung that measured approximately 2.1×3.0 cm in size, exhibiting lobules and burrs visible at the edge and pulling the pleura nearby. This mass was considered as left peripheral lung cancer with mediastinal and left hilar lymph node metastasis. And there were multiple ground glass nodules in both lungs, about 0.5-1.2 cm in diameter, with the larger ones being considered space-occupying lesions (**Figure 1A**).

The result of percutaneous lung puncture biopsy supported primary adenocarcinoma of the lung and immunohistochemistry analysis revealed positive staining for CKpan, EMA, KI-67 (approx. 10%), TTF-1, CK7, NapsinA, and negative staining for Ventana ALKD5F3. Due to limited lung biopsy samples, only EGFR gene testing was performed, and no mutation was found. Given the presence of metastatic lesions in the different left lobes, as well as mediastinal and ipsilateral hilar lymph node metastasis and contralateral lobe metastasis, she was diagnosed with stage IVA lung adenocarcinoma (cT4N2M1a).

The first-line treatment, consisting of pemetrexed + carboplatin + bevacizumab, was administered for 6 courses starting on August 22, 2019, and the treatment response was assessed as stable disease (SD) according to response evaluation criteria in solid tumors (RECIST). After maintenance treatment with pemetrexed + bevacizumab for 2 courses on January 9, 2020, the lesion in the superior lobe of the left lung and the enlarged lymph nodes of the left hilum were treated with radiation therapy from March 3 to April 20. After the radiation therapy, the CT scan reported that the lesion size was about 1.7×1.3 cm (**Figure 1B**).

The second-line treatment started on July 31, 2020, during which she complained of continuous dizziness and memory loss. The detection of an abnormal enhancement in the left frontal lobe and the posterior horn of the lateral ventricle on a brain magnetic resonance imaging scan on July 24 indicated disease progression with brain involvement (**Figure 1C**). This treatment consisted of six cycles of bevacizumab + pemetrexed + carboplatin + sintilimab. After two courses of treatment, no brain lesions were observed (**Figure 1D**), and a slight reduction in the size of their lung lesions was noted, resulting in an overall efficacy evaluation as a partial response (PR). Starting December 12th, the patient began maintenance treatment of pemetrexed + bevacizumab + sintilimab for six courses, after which the size of the lesion was about 1.7×1.1 cm and the efficacy was assessed as PR.

A Positron Emission Tomography-Computed Tomography (PET-CT) scan conducted on May 6, 2021 since she noticed a lump on the left neck, revealing metastasis in the left cervical, left submaxillary lymph nodes, and adrenal glands. The right adrenal gland exhibited a mass with a diameter of 1.9 cm, and the left one was full, which was considered tumor progression (**Figure 1E**). The third-line treatment consisted of 6 courses of albumin-bound paclitaxel + cisplatin starting on May 8, and after 4 courses of treatment, the overall efficacy was evaluated as PR and the lung lesion size was about 1.7×1.1 cm.

On December 11, 2021, a CT scan showed an enlargement of the lesion in the superior lobe of the lung that measured approximately 4.1×3.6 cm (**Figure 1F**), indicating further tumor progression. For the fourth-line treatment, local radiofrequency ablation could not be performed due to the lesion’s proximity to blood vessels. Consequently, the patient underwent 6 courses of gemcitabine + bevacizumab starting on December 14.

A new reassessment through a PET-CT scan on May 15, 2022 disclosed that the size of the lesion in the lung was about 6.5×4.4 cm with increasing metabolic activity, and there were 1.6 cm and 1.8 cm masses in the left and right adrenal glands, respectively (**Figure 1G**). The patient underwent fifth-line treatment from May 16 to June 17, which involved the administration of anlotinib at a dose of 12 mg once daily (2 weeks on/1 week off), in conjunction with palliative radiation therapy targeting the left submaxillary lymph nodes, pulmonary lesions, and bilateral adrenal metastatic lesions. By June 29, the pulmonary lesion was approximately 6.6×4.3 cm. In August, a second gene testing was conducted using targeted deep sequencing with a 1021-gene panel (Gene+) which extracted from peripheral blood samples and identified the BRCA2 gene p.L1908Rs*2 variant (germline mutation) without BRAF V600E-mutation. And the treatment was accordingly adjusted to the administration of olaparib starting on August 25, which dose was adjusted to 300mg/day divided into two doses orally due to myelosuppression. The patient was not regularly reexamined due to the COVID-19 pandemic.

**Figure 2:**
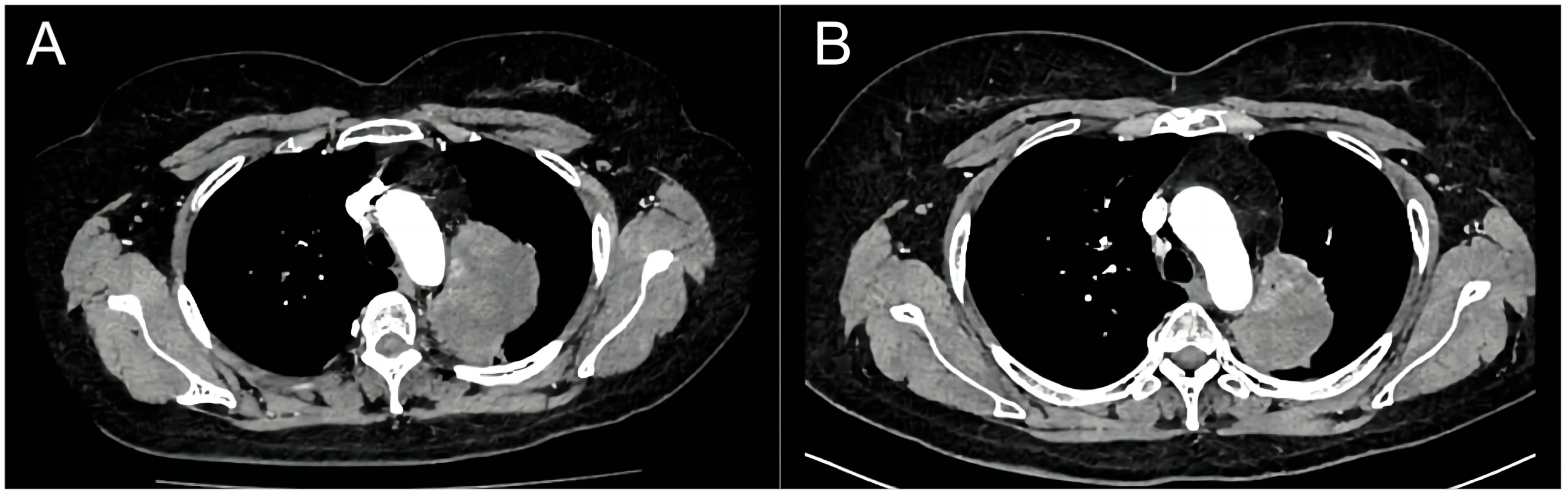
Computed tomography shows the comparison of lung lesions before **(A)** and after **(B)** the treatment of dabrafenib plus trametinib.

On February 24, 2023, the patient underwent a reevaluation, and the PET-CT scan showed new metastases in the right renal para-vascular lymph nodes and para-aortic lymph nodes. There was an increase in the size of the bilateral adrenal lesions (left: 3.9 cm, right: 4.7 cm), and the lung lesion measured approximately 6.6×5.2 cm (**Figure 1H**). Subsequently, the treatment of olaparib was ceased and the sixth-line treatment started on February 28, involving a regimen of albumin-bound paclitaxel + bevacizumab administered over 6 courses, resulting in an assessment of SD (**Figure 2A**).

Finally, in July 2023, the presence of the BRAF V600E mutation was confirmed through next-generation sequencing analysis by NGS panel spanning 769 cancer-related genes using peripheral blood samples during the third gene detection. As a result, a dual therapy approach involving dabrafenib 150 mg twice daily and trametinib 2mg once daily was implemented for the patient’s treatment. After only 2 treatment cycles, the lung lesion exhibited a rapid size reduction, delivering outstanding curative effects with no observable side effects. On November 5, the latest reexamination revealed that the size of the lung lesion was measured approximately 6.4×3.7 cm, and the right adrenal nodule reduced in size compared to previous assessments, while the left one displayed no significant changes (**Figure 2B**). The patient is currently still undergoing regular targeted treatment and follow-up care.

## 3 Discussion

In this article we report a clinical case of a patient with advanced-stage NSCLC with BRAF V600E mutation and germline BRCA2 mutation whose disease was refractory to several lines of therapy before the gene mutations were detected, she finally experienced a significant and successful therapeutic benefit from the combination treatment of dabrafenib and trametinib.

In this case, the patient underwent genetic testing three times as previously described. Due to the plasticity of the tumor, its molecular profile constantly changes during treatment, with drug-sensitive cells being eradicated and drug-resistant tumor cells continuing to clone. Screening for newly genetic mutations can inform the choice of subsequent treatments. The patient had no prior BRAF mutation detected, yet achieved an objective response to the front-line treatments, including radiotherapy, chemotherapy, and immunotherapy. However, the treatment effect became relatively poor after multiple lines of treatment, which was assessed as SD. Potential presence of other undetected resistance mechanisms may contribute to rapid progression and the second and third genetic analysis identified of germline mutation in BRCA2 and BRAF V600E mutation, providing a basis for the patient’s subsequent precision treatment, which emphasizes the importance of ongoing and dynamic monitoring of gene status. The first genetic detection using lung tissue samples was restricted to the EGRF gene due to limited tissue availability, while the subsequent two genetic detections were performed on blood samples. As the need for molecular testing with limited biopsy materials grows, repeatable liquid biopsy constitutes a promising noninvasive complement or alternative to traditional tissue biopsy for diagnosis and monitoring. The evaluation of circulating tumor cells (CTC) or circulating tumor DNA (ctDNA) in blood samples through minimally invasive techniques provides the necessary genetic material for comprehensive tumor mutation analysis. This approach not only produces significant clonal heterogeneity but also facilitates longitudinal monitoring of genetic status by doctors during targeted treatment, aiding in the prediction of drug resistance and disease progression (13, 14). However, there is currently no conclusive evidence demonstrating that liquid biopsy can completely replace the tissue samples in terms of sensitivity. With regard to mutation testing, false-negative BRAF results are possible due to limitations in genetic screening technologies. Thus, liquid biopsy and tissue genetic analysis complement each other, moreover, the importance of periodic testing should also be prioritized to better avoid false negatives. Besides, reasonable selection of methods and detection platforms also plays important roles in the accuracy of precision medicine (15). However, repeated and periodic genetic testing was difficult to practice in this case. During the course of the disease, we highly recommend the patient perform genetic testing using cancer tissue or blood sample every time disease progressed, which was declined due to invasive reason of needle biopsy and financial reason. And this indeed became a major obstacle to effective treatment and therefore extended treatment cycle.

During the fifth-line treatment, the patient agreed to the second genetic test using targeted deep sequencing with a 1021-gene panel (Geneplus-Beijing Institute), which collected peripheral blood samples and detected a pathogenic germline mutation in BRCA2 and negative mutation in BRAF, and this genetic testing has proven of value in the practice of clinical medicine (16). As a key component of transcriptional regulation and DNA repair, BRCA has been associated with clinical biomarkers for sensitivity to PARP inhibitors in breast, ovarian, and prostate cancers. In theory, PARP inhibitors are effective against any cancer cells with defects in HR, and BRCA1/2 genes are often found mutated or deleted in NSCLC (12). Nevertheless, uncertainties still exist regarding the underlying mechanisms of carcinogenesis of BRCA mutation in NSCLC and therapeutic efficacy of PARP inhibitors in HR-deficient NSCLC. Preclinical data revealed that BRCA1/2 knockout cells were significantly more sensitive to olaparib than wild-type cells (12). Several published case reports described the improved clinical benefits of PARP inhibitors for metastatic NSCLC patients with BRCA1/2 mutation, reporting PFS ranged from 5 to 13.5 months (17–20). In a single-center retrospective study, 6 NSCLC patients harbored pathogenic germline BRCA mutations and received PARP inhibitors. Among these, 4 patients exhibited enduring responses with a median PFS of 13 months (ranging from 6 to 36 months), of whom 2 patients were able to achieve sustained remissions for 3 years. These findings highlighted the high sensitivity of NSCLC with pathogenic BRCA mutations to PARP inhibitors, suggesting the potential tumorigenic effects of BRCA mutations in NSCLC (21). However, the above analysis on a small scale inevitably led to limitations of application. Unfortunately, the administration of olaparib showed poor efficacy in our case. After 6 months of medication, her disease progressed again. It is essential to conduct future studies with larger sample sizes to thoroughly investigate the efficacy of PARP inhibitors in the NSCLC treatment.

When the patient finished the sixth-line chemotherapy, the best response is SD. The suggestion of the third genetic detection was accepted due to her worry of the recurrence. Blood sample was obtained and comprehensive genomic profiling was performed by NGS using a 769 cancer-related gene panel (MinerVa®), which has been widely used in clinical trials (22). Fortunately, it demonstrated BRAF V600E mutant, providing further valuable clues for the precise treatment. In terms of BRAF-targeted therapy, the phase 2 trial (BRF113928) of dabrafenib and trametinib provided the first long-term survival data for patients with metastatic NSCLC harboring previously untreated BRAF V600E. After a minimum 5-year follow-up period of stage IV NSCLC patients with BRAF V600E, who had previously received 1 to 3 lines of treatments, the dual therapy of dabrafenib with trametinib as a treatment strategy yielded notable results: the objective response rate (ORR) was 68.4% (95% confidence interval (CI): 54.8-80.1), with a median disease control rate (DOR) of 9.8 months (95% CI: 6.9-18.3), a median PFS of 10.2 months (95% CI: 6.9-16.7), and a median OS of 18.2 months (95% CI: 14.3-28.6). For newly diagnosed stage IV NSCLC with BRAF V600E, the dual therapy of dabrafenib with trametinib also displayed a significant efficacy: the ORR was 63.9% (95% CI: 46.2-79.2), the DOR was 10.2 months (95% CI: 8.3-15.2), the median PFS was 10.8 months (95% CI: 7.0-14.5), and the median OS was 17.3 months (95% CI: 12.3-40.2). These findings show that the dual-target therapy of dabrafenib plus trametinib created an unprecedented response and survival benefit whether the patient had undergone prior treatment or not (11). Our case further reinforces the substantial curative effects of dabrafenib combined with trametinib following the discovery of the BRAF V600 mutation, highlighting the effectiveness of this combination strategy, regardless of the line of treatment.

A particular area of interest in this case is whether BRAF mutation was subsequently acquired and related to the utilization of olaparib. On the one hand, it is difficult to confirm the sequence of genetic mutations of BRFA and BRCA since there was a possibility of false negatives of genetic testing because of low sensitivity of liquid biopsy. On the other hand, there was case of acquired mutation of BRFA. A study showed that RAS/MAPK signaling was upregulated after the induction of PARP inhibitor resistance in PARP inhibitor-sensitive cells with wild-type RAS (23), and it also proposed that MEK inhibitors might restore sensitivity to PARP inhibitors (23), which showed a correlation between the olaparib and the upregulation of the MAPK pathway. However, this speculation still needs to be verified by further cellular and molecular trials. Conducting genetic testing at the point of disease progression during olaparib treatment could have provided crucial information for implementing a targeted treatment strategy at an earlier stage.

In conclusion, we reported a rare case of BRAF V600E mutated lung adenocarcinoma with BRCA2 germline mutation. This case report emphasizes the crucial role of ongoing monitoring of genetic mutations and repeat genetic testing with more extensive genetic panels during patient management, which help facilitate the translation of precision medicine into clinical practice and provide optimized and tailored treatment for genetically matched patients. It also underscores the effectiveness of the combination strategy of dabrafenib plus trametinib, regardless of the prior number of treatment lines. Since only one case of lung adenocarcinoma with BRAF V600E and BRCA2 germline mutation has been observed in this report, the clinical data remains very limited, requiring additional research to identify the patient subgroups most likely to experience clinical benefits from PARP inhibitors and possible influence on subsequent molecular mechanism in this population.

## Data availability statement

The datasets presented in this study can be found in online repositories. The names of the repository/repositories and accession number(s) can be found in the article/supplementary material.

## Ethics statement

The studies involving humans were approved by the Ethics Committee of the First Hospital of Jilin University. The studies were conducted in accordance with the local legislation and institutional requirements. The human samples used in this study were acquired from the patient requested genetic testing. Written informed consent for participation was not required from the participants or the participants’ legal guardians/next of kin in accordance with the national legislation and institutional requirements. Written informed consent was obtained from the individual(s) for the publication of any potentially identifiable images or data included in this article.

## Author contributions

HZ: Writing – original draft. XC: Writing – original draft. JY: Writing – review & editing. CC: Writing – review & editing. ZL: Writing – review & editing.
